# Association of DPP-4 Concentrations with the Occurrence of Gestational Diabetes Mellitus and Excessive Gestational Weight Gain

**DOI:** 10.3390/ijms25031829

**Published:** 2024-02-02

**Authors:** Magdalena Niebrzydowska-Tatus, Aleksandra Pełech, Katarzyna Bień, Julia Mekler, Miracle Santiago, Żaneta Kimber-Trojnar, Marcin Trojnar

**Affiliations:** 1 Chair and Department of Obstetrics and Perinatology, Medical University of Lublin, 20-090 Lublin, Poland; mniebrzydowska7@gmail.com (M.N.-T.); apilszyk@gmail.com (A.P.); 2 Student’s Scientific Association at the Chair and Department of Obstetrics and Perinatology, Medical University of Lublin, 20-090 Lublin, Poland; kaasiabien@gmail.com (K.B.); julia.mekler@yahoo.com (J.M.); msantiago.mul@gmail.com (M.S.); 3 Chair and Department of Internal Diseases, Medical University of Lublin, 20-059 Lublin, Poland; marcin.trojnar@umlub.pl

**Keywords:** DPP-4, gestational diabetes mellitus, excessive gestational weight gain, bioelectrical impedance analysis

## Abstract

Gestational diabetes mellitus (GDM) is considered one of the most common diseases that occur during pregnancy. In addition to increasing the risk of numerous complications throughout gestation, it is also believed to have a long-term potential to impact the risk of developing type 2 diabetes mellitus (T2DM) and cardiovascular disease for the mother and her offspring. While there are clear guidelines for healthy weight gain in pregnancy depending on pre-pregnancy BMI, as well as dietary and training recommendations to achieve this, an increasing number of women are experiencing excessive gestational weight gain (EGWG). Such patients have a higher risk of developing GDM and gestational hypertension, as well as requiring caesarian delivery. Dipeptidyl peptidase-4 (DPP-4) is a glycoprotein that seems to play an important role in glucose metabolism, and inhibition of its activity positively affects glucose regulation. The aim of our study was to compare DPP-4 concentrations in patients with GDM and EGWG with healthy women. DPP-4 levels were assessed in serum and urine samples collected on the day of delivery. The bioelectrical impedance analysis (BIA) method was also used to analyze the body composition of patients on the second day of the postpartum period. DPP-4 serum concentrations were significantly higher in patients in the GDM and EGWG groups compared to healthy women. Urinary DPP-4 concentrations were significantly higher in the control and GDM groups than in the EGWG group. Serum DPP-4 levels were positively correlated with BMI measured before pregnancy, on the delivery day, and in the early postpartum period, among other factors. According to our knowledge, this is the first study to determine DPP-4 levels in EGWG patients. DPP-4 may be related to the occurrence of GDM and EGWG; however, this requires further research.

## 1. Introduction

Gestational diabetes mellitus (GDM) is defined as glucose intolerance with onset—or first detection—occurring during pregnancy. GDM accounts for 90% of all diabetes mellitus (DM) cases among pregnant women [1], affecting over one-sixth of pregnancies worldwide, making it the most common complication in pregnancy [2]. Risk factors include high body mass index (BMI) and obesity, advanced maternal age, a family history of any form of diabetes, and a maternal history of insulin resistance [3].

Though the biological mechanisms responsible for GDM have not yet been fully elucidated, the pathophysiology is thought to follow the same etiology as type 2 diabetes mellitus (T2DM), with the physiological changes in pregnancy causing increased susceptibility to glycemic dysregulation. The increased metabolic demand of pregnancy causes blood glucose to rise and insulin sensitivity to fall, and pancreatic β-cells subsequently undergo hyperplasia and hypertrophy [3]. As an adaptive mechanism to facilitate the transfer of nutrients from the mother to the developing fetus, some degree of insulin resistance occurs during a healthy pregnancy [4]. In GDM, however, β-cells fail to compensate for the increased glucogenic demands of pregnancy, resulting in hyperglycemia.

Dysfunction in insulin signaling during pregnancy, which can result in chronic insulin resistance, is also thought to play a role in GDM; clinical research has found that the rate of insulin-stimulated glucose uptake in GDM patients is less than half of that in normoglycemic pregnant patients. In addition to insulin, dysregulation of other hormones is also associated with GDM, e.g., while a normal pregnancy confers some degree of leptin resistance, it is increased in GDM, resulting in hyperleptinemia. Accumulation of adipose tissue during pregnancy may also contribute to dysglycemia via increased adipose tissue macrophages, which secrete proinflammatory cytokines that impair insulin signaling and inhibit the release of insulin from β-cells [3].

Weight gain in pregnancy is a physiological process in response to the development of the fetus, that includes body composition, as well as the weight of the fetus, placenta, and amniotic fluid [5]. It is also an important predictor of a healthy pregnancy and delivery outcomes [6]. The Institute of Medicine (IOM) has provided guidelines for recommended weight gain in pregnancy depending on the pre-pregnancy BMI. As follows: 12.5–18 kg for underweight women (BMI < 18.5), 11.5–16 kg for normal weight women (BMI 18.5–24.9), 7–11.5 kg for overweight women (BMI 25.0–29.9), and 5–9 kg for obese women (BMI ≥ 30.0) [7]. There are many factors that contribute to excessive gestational weight gain (EGWG); however, dietary habits and preconception weight seem to be the most important [6,8]. Despite the increased risk of pregnancy-related complications in women with pre-pregnancy obesity and EGWG, it appears that they are also more common in women with normal pre-pregnancy BMI and EGWG, which suggests that EGWG might be an independent risk factor of adverse outcomes [9]. Women with EGWG are more likely to develop GDM, gestational hypertension, prolonged second stage of delivery, postpartum hemorrhage, and spontaneous preterm labor [6,10,11].

Dipeptidyl peptidase-4 (DPP-4) is a glycoprotein expressed on the surface of most cell types, playing a role in a myriad of cellular functions, including immune regulation, apoptosis, and glucose metabolism. The enzymatic role of DPP-4 is as a serine exopeptidase, cleaving N-terminus “shields” from the ends of various polypeptides; in this function, DPP-4 can halt the action of a wide variety of substrates, including growth factors, chemokines, neuropeptides, and vasoactive peptides [12]. Clinically, the most important role of DPP-4 is in glucose metabolism, where it binds and degrades incretins—i.e., hormones that regulate postprandial insulin release. DPP-4 inhibition leads to greater bioavailability of these incretins, including glucagon-like peptide-1 (GLP-1), which prolongs insulin action. DPP-4 inhibitors (DPP-4i), a class of drugs called gliptins, act as oral hypoglycemics by extending incretin activity—promoting insulin secretion, suppressing glucagon, and slowing gastric emptying [13].

While the role of DPP-4 in the pathogenesis of GDM has not been fully elucidated, it is considered to be of significant importance—both in maternal dysglycemia and in developing the glucose metabolism of the fetus. Studies have shown that plasma DPP-4 is elevated in children born to mothers who are obese during pregnancy, with animal models revealing that DPP-4i can prevent the development of obesity in offspring [14]. Clinical trials studying the effect of DPP-4 inhibitors on GDM have demonstrated a reduction in insulin resistance, alleviation of the symptoms associated with hyperglycemia, reduction in fasting plasma glucose and serum insulin, and downregulation of a biomarker indicative of glucose intolerance [15]. While more research is needed to understand the role of DPP-4 in GDM, the existing literature suggests that DPP-4i (i.e., gliptin) therapy could help mitigate the capacity of GDM to program the fetus for future obesity and metabolic disease [16]. A systematic review [2] concluded that gliptins helped normalize blood glucose, reduced insulin resistance, enhanced β-cell function, and reduced the rate of postpartum diabetes among GDM patients.

### 1.1. Use of Bioelectrical Impedance Analysis in Postpartum Women

Excessive adiposity in pregnancy is strongly associated with an altered metabolic profile, which is linked to adverse outcomes in both mother and child [17], with EGWG and postpartum weight retention (PPWR) contributing significantly to long-term adverse health outcomes [18]. Bioelectrical impedance analysis (BIA) is a noninvasive technique for assessment of body composition. BIA is a reliable measure, despite the challenges of assessing a regularly fluctuating perinatal and postpartum BMI. BIA measures electrical resistance across various body tissues (e.g., fat, muscle) in relation to the electrical conductivity of body fluid volume. It can be used as a prognostic indicator for gestational and postpartum outcomes, particularly in clinical assessment of EGWG and PPWR and the corresponding adiposity-related risks of obstetric and postpartum complications and should be subsequently considered as a reliable screening tool [19].

### 1.2. Phenomenon of Maternal Programming

The maternal programming hypothesis theorizes that adverse effects early in fetal development lead to permanent changes in the offspring’s morphology, physiology, and metabolism. In addition to programming affecting the offspring, GDM is associated with a myriad of obstetric complications and poor postpartum health outcomes. GDM pregnancies are more likely to result in gestational hypertension, including preeclampsia, polyhydramnios, preterm premature rupture of membranes, preterm delivery, and delivery requiring cesarean section [20]. While normal glucose levels are usually reestablished in the postpartum period, GDM patients are more vulnerable to developing T2DM later in life [21], with studies revealing GDM patients to be >7 times more likely than women who had normoglycemic pregnancies. With about half of these patients developing diabetes within 10 years postpartum, GDM is one of the strongest risk factors for T2DM [22]. Women with GDM also experience a two-fold risk of cardiovascular and cerebrovascular events, independent of postpartum T2DM, in the first decade after pregnancy. This may be linked to its association with gestational hypertension, which increases the risk of long-term metabolic and vascular disease, including chronic hypertension, ischemic heart disease, ischemic stroke, and myocardial infarction [23]. In addition to complications during pregnancy and delivery, EGWG can also cause long-term effects by increasing the risk of maternal obesity due to postpartum weight retention [8,10].

**Figure 1:**
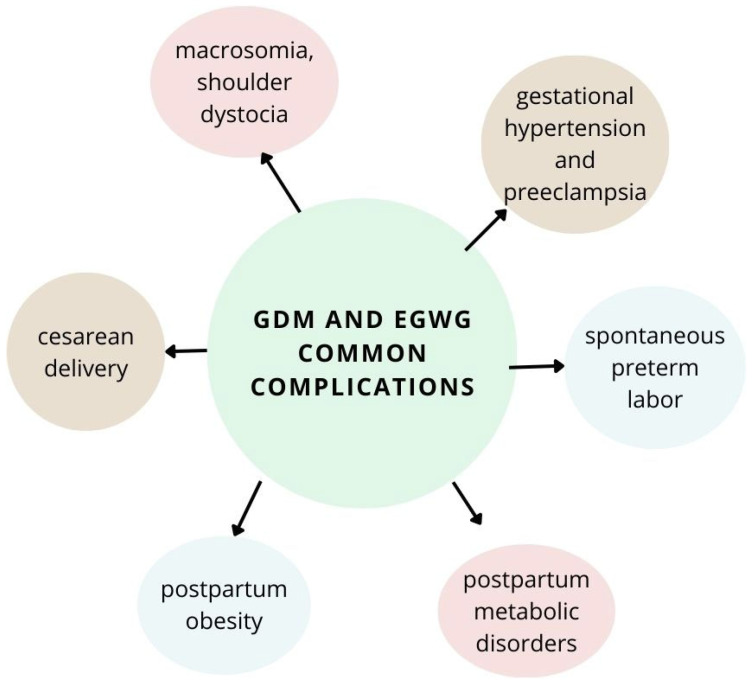
Common complications of GDM and EGWG.

Common complications for GDM and EGWG are presented in Figure 1.

The aim of our study was to further investigate the relationship between DPP-4 concentrations with the occurrence of GDM and EGWG and to correlate them with clinical parameters. We hypothesized that DPP-4 serum and urine levels would be higher in GDM and EGWG groups compared to healthy controls. We also hoped to reveal some associations between DPP-4 concentrations and metabolic parameters.

## 2. Results

Our study included a total of 74 patients who were divided into three groups. The GDM group involved 25 women who had at least one abnormal OGTT measurement between 24 and 26 weeks of pregnancy. They were treated with diet alone and their pre-pregnancy BMI was the highest of all groups. The EGWG group enrolled 25 women whose pre-pregnancy BMI was normal, but according to IOM criteria [8], their gestational weight gain was excessive (mean 24.44 kg, SD 2.62 kg). Patients in this group did not meet the criteria for a diagnosis of GDM. The control group included 24 women with normal pre-pregnancy BMI and normal gestational weight gain along with no GDM diagnosis. The age range of patients in all groups was 22–35 years old. A comparison of characteristics of the study subjects is presented in Table 1.

### 2.1. Comparison of DPP-4 Concentration in GDM Patients, EGWG Patients, and Healthy Controls

Comparing DPP-4 concentrations in serum on delivery day and urine tests on the same day, there were statistically significant differences (*p* > 0.05) between mothers with GDM, patients with EGWG, and controls (Table 2).

### 2.2. Correlations of DPP-4 Determinations in Serum and Urine in Delivery Day

#### 2.2.1. All Groups

A statistically insignificant relationship was found (*p* > 0.05).

#### 2.2.2. GDM Group

A statistically insignificant relationship was found (*p* > 0.05).

#### 2.2.3. EGWG Group

A statistically insignificant relationship was found (*p* > 0.05).

#### 2.2.4. Control Group

A statistically significant relationship was found (*p* < 0.05).

**Figure 2:**
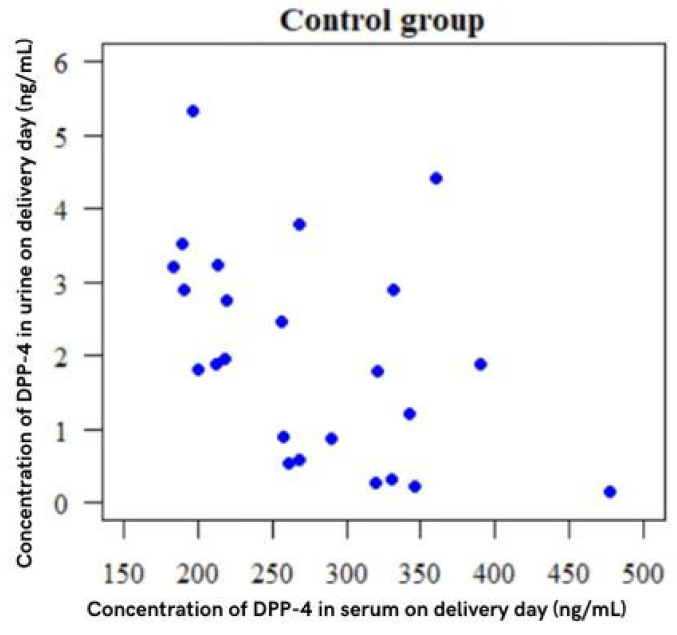
Spearman rank correlation coefficient = *p* = 0.013; *p* < 0.001.

The correlation is negative, so the higher the serum DPP-4 concentration, the lower the concentration in urine, and vice versa: the higher the concentration in urine, the lower the concentration in serum (Figure 2).

### 2.3. Correlations with DPP-4 Concentrations in All Groups

Correlations of selected parameters with DPP-4 concentrations in all groups are presented in Table 3.

## 3. Discussion

GDM is one of the most common diseases that occur during pregnancy and has long-term effects on the mother and her offspring by increasing the risk of developing metabolic diseases in the future. GDM is a condition of glucose intolerance diagnosed for the first time during pregnancy without a known history of glucose metabolism disorders before [16,24]. EGWG is diagnosed by exceeding the recommended weight gain depending on pre-pregnancy weight according to the IOM 2009 criteria [7]. Its occurrence is associated with numerous complications, including the development of GDM [10].

EGWG has become an increasingly common problem. A meta-analysis by Goldstein et al. [25] conducted on more than 1 million women found that 47% exceeded IOM recommendations in gestational weight gain, while 23% gained less weight than recommended. EGWG was associated with a higher risk of pregnancy complications compared to women with normal pregnancy weight gain. Another meta-analysis by Zhou et al. [11] found pre-pregnancy overweight, younger age, primiparity, and nicotinism to have the greatest impact on the development of EGWG. Interestingly, there is no association between the occurrence of EGWG and the level of education and access to dietary recommendations during pregnancy.

The aim of our research was to evaluate the potential association of DPP-4 levels with the occurrence of GDM and EGWG during pregnancy. Several studies have been conducted to assess the link of DPP-4 concentrations with the occurrence of GDM. However, to our knowledge, no one has yet evaluated the concentration of this molecule in women with EGWG observed. The pathogenesis of GDM is still not fully understood, but the most significant factor is an increase in insulin resistance, which eventually leads to persistent hyperglycemia, as in T2DM. The incretin system, which involves GLP-1 and glucose-dependent insulinotropic polypeptide (GIP), regulates these effects, while DPP-4 is a glycoprotein responsible for the degradation of the above-mentioned molecules [24]. Moreover, elevated DPP-4 activity is observed in obese patients, which may suggest its potential link to the pathogenesis of obesity [14].

We examined DPP-4 concentrations in serum and urine samples collected on the day of delivery in all patients’ groups. In addition, we performed bioelectrical impedance analysis (BIA) to assess the body composition of the patients on the second day after delivery.

**Figure 3:**
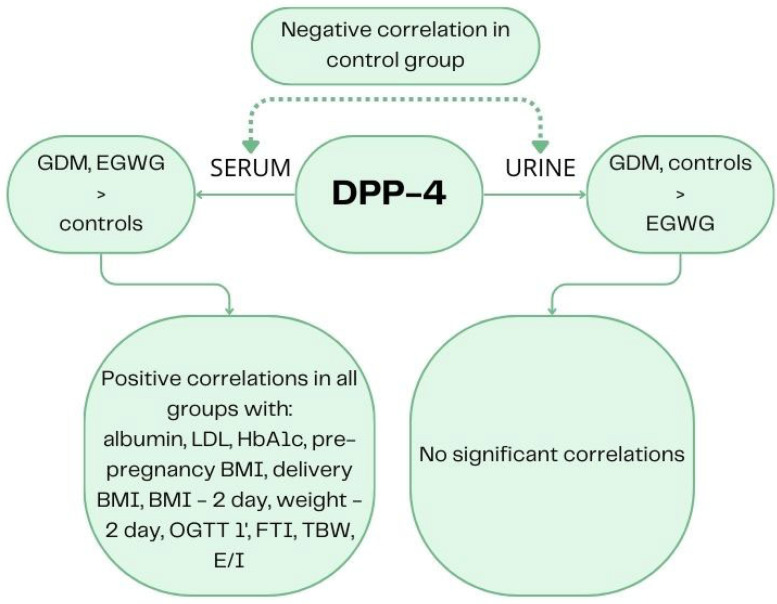
Graphical representation of the results.

Serum DPP-4 levels were significantly higher in the GDM and EGWG groups compared to healthy women, while urine levels appeared to be significantly increased in the control and GDM groups compared to the EGWG group. We also observed a negative correlation between serum and urine DPP-4 concentrations in the control group, with no significant relationship in the other groups (Figure 3).

Incretins are responsible for about 80% of total insulin secretion in response to oral glucose intake. They include GLP-1 and GIP, which are known for maintaining glucose homeostasis by decreasing glucagon release, slowing gastric emptying, and suppressing appetite, with additional positive effects on weight control [26,27]. GLP-1 agonists are used in T2DM treatment due to their beneficial impact on reducing BMI and HbA1c [2]. DPP-4 is a molecule that plays an important role in regulating metabolism, appetite, and body composition [14] and exerts its functions by degrading GLP-1, which subsequently reduces insulin secretion in response to glycemic levels. Elevated values of DPP-4 are observed in T2DM, while DPP-4 inhibitors, i.e., gliptins, are used for its treatment [28,29,30].

In GDM and EGWG, we can observe elevated levels of leptin, tumor necrosis factor-α (TNF-α), abnormal oxidative stress, and enhanced reactive oxygen species (ROS) generation. This leads to an imbalance between pro- and anti-inflammatory cytokines, resulting in low-grade inflammation, potentially related to the development of insulin resistance [28,31]. There is some proof that DPP-4 may mediate the above-mentioned processes, as a molecule that affects the regulation of chemokine and cytokine activity, including its action as a binding site for the C-X-C chemokine receptor type 4 (CXCR4) [14,32]. In obesity, an over-activation of DPP-4 is observed, resulting in the accumulation of ROS and the production of proinflammatory cytokines [14]. Potentially, increased DPP-4 activity in GDM and EGWG may influence the development of inflammation, impair insulin signaling, and, finally, contribute to insulin resistance [28]. Preclinical studies have shown that DPP-4 inhibitors can reduce inflammation [33], while Sun et al. [15] demonstrated that using the DPP-4 inhibitor, sitagliptin, improved glycemic control and insulin sensitivity in women with GDM.

GDM usually resolves spontaneously after delivery. However, women with GDM history are more likely to develop T2DM in the future [30]. The placenta appears to contribute to the pathogenesis of GDM as it affects insulin resistance progression, and its delivery during labor seems to improve glucose metabolism, which is reflected in the reduced demands for the insulin used to treat GDM shortly after delivery [28]. It is believed that dysfunction of the incretin system may have a role in the pathogenesis of GDM [2], while pharmacological treatment, regulating its function, has the potential to prevent or delay the development of T2DM in such patients [30].

There is growing evidence that increased DPP-4 activity can potentially influence excessive fat deposition and metabolic dysfunction, which can result in the development of insulin resistance and obesity [14,24]. Adipose tissue is not only a source of spare energy but also has some endocrine functions executed through the secretion of adipokines. Developing obesity can impair its activity, leading to an increased risk of T2DM. Elevated expression of DPP-4 in subcutaneous and visceral adipose tissue has been observed in obese patients in comparison to normal weight subjects, while its release is significantly associated with metabolic parameters such as BMI, waist circumference, triglycerides, and Homeostasis Model Assessment of Insulin Resistance (HOMA-IR) [34]. Our study seems to confirm these results, as we observed a significant correlation of serum DPP-4 levels with BMI measured before pregnancy, on delivery day, and in the early postpartum period. In addition, we showed a positive association of serum DPP-4 concentrations with weight measured on the second day after delivery, LDL, HbA1c, and OGTT 1’, as well as parameters determined with BIA, i.e., FTI, TBW, and E/I. We did not observe any significant associations between urinary DPP-4 levels and investigated parameters.

An increasing number of researchers are looking for a link between DPP-4 and the development of metabolic disorders in pregnancy. In a study by Montaniel et al. [14], elevated serum DPP-4 activity was shown in obese women. In our research, we also found increased serum DPP-4 levels in both GDM and EGWG groups compared to healthy women. Another research study by Kandzija et al. [28] evaluated DPP-4 activity in syncytiotrophoblast-derived extracellular vesicles (STB-EVs) in GDM patients and healthy women. They documented a secretion of STB-EVs binding enzymatically active DPP-4 in both groups; however, in the GDM group, it was 8-fold higher compared to healthy controls, which brings some questions about the placenta’s role in the pathogenesis of GDM.

However, some studies cannot confirm the described associations. Liu et al. [24] observed no significant differences between serum DPP-4 concentrations in healthy and GDM patients. The researchers suggest the existence of mechanisms unrelated to the incretin system being responsible for impaired glucose metabolism in GDM. They conducted correlation tests between HbA1c and serum DPP-4 concentration in the GDM group; however, it showed no association. On the contrary, our study revealed a positive relationship between serum DPP-4 concentrations and HbA1c in all patients.

Due to the possible impairment of the incretin system occurring in GDM, some researchers are studying the potential of pharmacotherapy targeting the incretin system in pregnant women with GDM and in the postpartum period. There are some interesting outcomes suggesting that administering DPP-4 inhibitors in the second trimester of pregnancy in women with GDM can significantly improve glucose metabolism [15]. A similar effect can be achieved by adding sitagliptin to metformin treatment in women with impaired glucose tolerance and recent history of GDM to prevent T2DM development [30].

There are few studies evaluating urinary DPP-4 levels in human urine in general, and to our knowledge, no one to date has evaluated its levels in the urine of pregnant women with GDM and EGWG. Sun et al. [35] showed an association of urinary DPP-4 levels with the severity of diabetic kidney disease, while another study observed elevated urinary DPP-4 levels in patients with T2DM [36]. Klein et al. [37] believe that urinary DPP-4 could potentially be used as a predictive biomarker of improved urine albumin-to-creatinine ratio (UACR) in response to linagliptin treatment in patients with diabetic kidney disease. We hypothesized that urine could serve as an easy-to-collect and non-invasive material as a potential marker for metabolic disorders. In our study, we showed significantly elevated urine DPP-4 levels in healthy and GDM women compared to the EGWG group. Interestingly, we also observed a negative correlation between serum and urine DPP-4; however, it only applied to healthy controls. We did not find any significant correlations between urine DPP-4 levels and metabolic parameters. All the patients in our study had normal renal function.

The main limitations of our study were the small size of the groups and only one determination of maternal serum and urine DPP-4 concentration on the delivery day. We believe it would be beneficial to investigate changes in DPP-4 levels in different pregnancy trimesters and correlate these values with metabolic parameters, such as BMI and glucose control features, to assess the impact of this molecule on the pathogenesis of GDM. Moreover, our research lacked the follow-up of patients for postpartum metabolic disorders and weight retention, which could help to identify the long-term impact of DPP-4 activity on maternal programming to T2DM and obesity in women with GDM and EGWG history. Not to mention, we lack the analysis of dynamics in DPP-4 concentrations shortly after delivery, which could be used to establish the relevance of the placenta in the regulation of the molecule’s activity. We believe there is an interesting direction for further research in the above-mentioned aspects.

## 4. Materials and Methods

The study comprised seventy-four Caucasian females in a singleton term pregnancy (after 37 weeks of gestation) who delivered at the Chair and Department of Obstetrics and Perinatology at the Medical University of Lublin. We divided the patients into three groups: the first group consisted of 25 women with GDM, the second group consisted of 25 women with excessive gestational weight gain, and the third group included 24 females with normal singleton term pregnancy without other metabolic abnormalities and risk factors. All patients included in our study could not have been diagnosed with other chronic or gestational diseases, which made the qualification process significantly more difficult, given that GDM often coexists with other conditions, such as gestational hypertension.

First, each pregnant woman was tested by a fasting plasma glucose test before 10 weeks of pregnancy, usually at the first examination during pregnancy. If the result of the test was less than 92 mg/dL, the 75 g oral glucose tolerance test (OGTT) was performed between 24 and 26 weeks of pregnancy in accordance with the Regulation of the Minister of Health, which has been in force in Poland since 1 January 2019 [38]. However, OGTT should be ordered at the first examination during pregnancy if a patient has a history of GDM, GDM risk factors like obesity or macrosomia, or the result of the fasting plasma glucose test before 10 weeks of pregnancy exceeds 92 mg/dL.

The GDM group included patients whose fasting blood glucose before 10 weeks of pregnancy was normal, but they had an abnormal OGTT result between 24 and 26 weeks of pregnancy. At least one of the measurement results was in the following ranges: fasting blood glucose ≥ 92 mg/dL, at 60 min ≥ 180 mg/dL, and at 120 min ≥ 153 mg/dL, which allowed us to diagnose GDM. Considering we only qualified patients with normal glycemic control with dietary treatment alone, women with pre-pregnancy overweight were also included. The Polish Diabetes Association does not recommend treatment with oral antidiabetic drugs at this point [39].

The EGWG group included patients whose blood glucose before 10 weeks of pregnancy and OGTT between 24 and 26 weeks of pregnancy were normal, as well as their pre-pregnancy BMI (18.5–24.9), while their gestational weight gain did not meet the 2009 IOM criteria and exceeded 16 kg [7].

Women who were not diagnosed with GDM and had normal pre-pregnancy BMIs, as well as normal gestational weight gain (11.5–16 kg), were categorized as controls.

**Figure 4:**
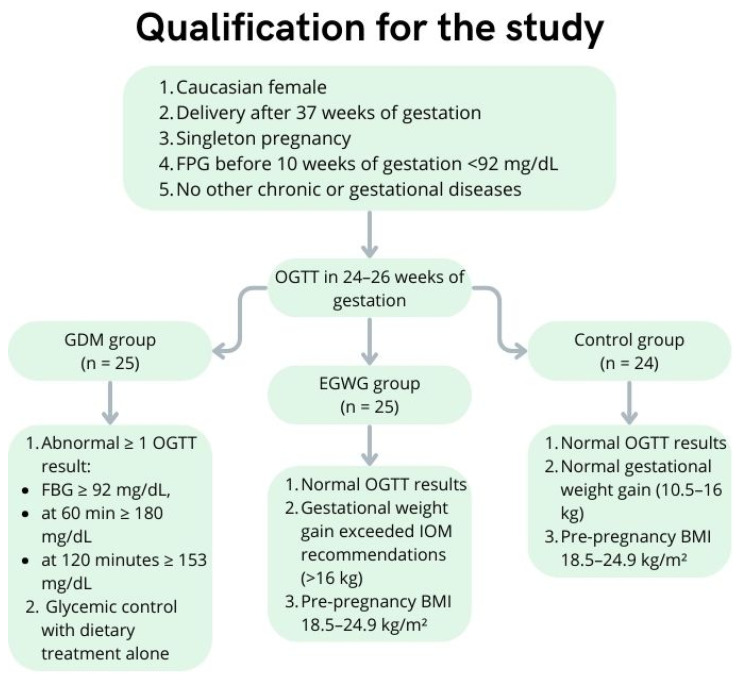
Qualification for the study.

Eligibility criteria are summed up in Figure 4.

All patients were informed about the study protocol, and detailed written consent was obtained from each patient who agreed to participate in the study. The study protocol received approval from the Bioethics Committee of the Medical University of Lublin (KE-0254/61/2020, approved on 26 March 2020).

Many parameters were measured in our study in serum and urine, including albumin, total cholesterol, high-density lipoprotein cholesterol (HDL), low-density lipoprotein cholesterol (LDL), triglycerides, hemoglobin A1c (HbA1c), and mean corpuscular volume (MCV). We also monitored BMI parameters (pre-pregnancy, on the day of delivery, and on day 2 postpartum) as an adequate indicator of body weight gain.

In the early postpartum period (i.e., 48 h after delivery), we used the bioelectrical impedance analysis (BIA) method and a body composition monitor (BCM) (Fresenius Medical Care) to measure maternal body composition and hydration status.

DPP-4 concentrations were determined in maternal serum and urine samples taken on the day of delivery using an enzyme-linked immunosorbent assay (Sandwich ELISA) and kits available on the market (R&D Systems, Inc., Minneapolis, MN, USA; Quantikine Human DPP-4 Immunoassay; catalog number DGAL90; detection range 0.2–10 ng/mL, sensitivity 0.028 ng/mL).

In our study, we used the chi-squared test (with Yates correction for 2 × 2 tables) or the Fisher exact test (in case of low expected values) for comparisons of qualitative variables between groups. The Mann–Whitney test was used for comparisons of quantitative variables between two groups. Spearman’s correlation coefficient was used to assess the correlation between two quantitative variables. The significance level was set to 0.05. All the analyses were conducted in R software, version 4.3.1.

## 5. Conclusions

Our study revealed significantly higher serum DPP-4 levels in the GDM and EGWG groups compared to healthy women, as well as their correlation with metabolic parameters. Urinary DPP-4 concentrations were significantly elevated in the control and GDM groups compared to the EGWG group, while there was no link between their levels and metabolic parameters. We also found a negative correlation between serum and urine DPP-4 levels, but it was only applicable to the control group. Nevertheless, we do not perceive urine as a reliable diagnostic tool for assessing DPP-4 levels in pregnant women.

Based on the above results, we believe there might be a potential link between elevated serum DPP-4 levels and the occurrence of GDM and EGWG. However, more studies on larger groups of patients are needed to assess its utility as a marker of metabolic disorders, as well as its potential association with the development of GDM. Hopefully, it could lead us to a better understanding and management of the diseases.

## Figures and Tables

**Table 1:** Comparison of characteristics of the study subjects.

Variables		GDM Group n = 25 A	EGWG n = 25 B	Control Group n = 24 C	*p*
Albumin [mg/mL]	Mean (SD)	3.49 (0.25)	3.58 (0.25)	3.73 (0.15)	*p* = 0.002 *
					C > B, A
Total cholesterol (mg/dL)	Mean (SD)	222.9 (36.08)	222.24 (40.19)	225.62 (35.63)	*p* = 0.904
	Range	176–312	164–313	176–287	
HDL (mg/dL)	Mean (SD)	64.76 (9.04)	64.76 (16.13)	64.76 (11.52)	*p* = 0.039 *
					C > A
LDL (mg/dL)	Mean (SD)	115.52 (26.27)	111.96 (27.35)	112.08 (27.55)	*p* = 0.784
Triglycerides (mg/dL)	Mean (SD)	204.04 (52.8)	210.2 (55.21)	160.79 (28.83)	*p* < 0.001 *
					A, B > C
HgbA1c (IFCC) (mmol/mol)	Mean (SD)	5.42 (0.35)	5.34 (0.33)	5.12 (0.29)	*p* = 0.011 *
					A, B > C
MCV [fl]	Mean (SD)	89.90 (3.41)	87 (2.57)	87.78 (4.78)	*p* = 0.009 *
					A > B, C
Pre-pregnancy BMI [kg/m^2^]	Mean (SD)	26.46 (3.6)	22.55 (1.49)	22.24 (2.15)	*p* < 0.001 *
					A > C, B
OGTT-0’ [mmol/L]	Mean (SD)	93.64 (5.11)	78.62 (4.92)	79.83 (4.02)	*p* < 0.001 *
					A > C, B
OGTT-1’ [mmol/L]	Mean (SD)	175.16 (13.92)	124.82 (23.32)	126.5 (20.8)	*p* = 0.011 *
					A, B > C
OGTT-2’ [mmol/L]	Mean (SD)	154.64 (26.56)	104.41(16.62)	102.96 (20.65)	*p* < 0.001 *
					A > B, C
Delivery BMI (kg/m^2^)	Mean (SD)	30.69 (3.85)	31.12 (3.66)	26.52 (3.29)	*p* < 0.001 *
					B, A > C
BMI—2 day (kg/m^2^)	Mean (SD)	28.87 (3.87)	28.52 (2.52)	24.19 (2.87)	*p* < 0.001 *
					A, B > C
Weight—2 day (kg)	Mean (SD)	79.25 (11.88)	79.63 (9.92)	65.97 (6.86)	*p* < 0.001 *
LTI (kg/m^2^)	Mean (SD)	12.22 (1.23)	13.28 (1.43)	12.23 (1.6)	*p* < 0.05
					B > C, A
FTI (kg/m^2^)	Mean (SD)	16.11 (3.9)	14.76 (2.68)	11.87 (2.07)	*p* < 0.001 *
					A, B > C
TBW (%)	Mean (SD)	34.15 (2.74)	36.81 (3.81)	31.96 (3.19)	*p* < 0.001 *
					B > A, C
E/I	Mean (SD)	0.97 (0.08)	0.94 (0.08)	0.90 (0.07)	*p* = 0.007 *
					A, B > C
BCM (kg)	Mean (SD)	17.91 (2.46)	21.13 (3.65)	19.26 (3.56)	*p* = 0.005 *
					B > A

*p*—Qualitative variables: Kruskal–Wallis test + post hoc analysis (Dunn test); SD—standard deviation; *—difference statistically significant (*p* < 0.05); A—group of patients with gestational diabetes mellitus; B—group of patients with excessive gestational weight gain; C—control group of patients; GDM—gestational diabetes mellitus; EGWG—excessive gestational weight gain; HDL—high-density lipoprotein cholesterol; LDL—low-density lipoprotein cholesterol; HbA1c—hemoglobin A1c; MCV—mean corpuscular volume; OGTT—oral glucose tolerance test; BMI—body mass index; LTI—lean tissue index; FTI—fat tissue index; TBW—total body water; E/I—extracellular water to intracellular water index; BCM—body cell mass.

**Table 2:** Comparison of DPP-4 concentration.

Parameter		GDM Group n = 25 A	EGWG n = 25 B	Control Group n = 24 C	*p*
Concentration of DPP-4 in serum on delivery day (ng/mL)	Mean (SD)	344.5 (67.87)	323.85 (58.34)	276.96 (75.45)	*p* = 0.003
					A, B > C
Concentration of DPP-4 in urine on delivery day (ng/mL)	Mean (SD)	3.4 (3.96)	0.94 (0.75)	2.04 (1.44)	*p* = 0.007
					C, A > B

*p*—Kruskal–Wallis test + post hoc analysis (Dunn test); SD—standard deviation; A—group of patients with gestational diabetes mellitus; B—group of patients with excessive gestational weight gain; C—control group of patients; GDM—gestational diabetes mellitus; EGWG—excessive gestational weight gain.

**Table 3:** Correlation with DPP-4 concentrations in all groups.

	Concentration of DPP-4 in Serum on Delivery Day (ng/mL)	Concentration of DPP-4 in Urine on Delivery Day (ng/mL)
Albumin	r = −0.25, *p* = 0.032 *	r = 0.16, *p* = 0.174
Total cholesterol (mg/dL)	r = 0.211, *p* = 0.072	r = −0.015, *p* = 0.9
HDL (mg/dL)	r = 0.035, *p* = 0.766	r = 0.083, *p* = 0.481
LDL (mg/dL)	r = 0.242, *p* = 0.038 *	r = −0.109, *p* = 0.354
TG (mg/dL)	r = 0.072, *p* = 0.544	r = −0.067, *p* = 0.569
HgbA1c (%)	r = 0.238, *p* = 0.041 *	r = −0.038, *p* = 0.749
MCV (fl)	r = 0.006, *p* = 0.956	r = 0.162, *p* = 0.167
Pre-pregnancy BMI (kg/m^2^)	r = 0.347, *p* = 0.002 *	r = 0.197, *p* = 0.092
OGTT-0’	r = 0.061, *p* = 0.604	r = 0.157, *p* = 0.182
OGTT-1’	r = 0.388, *p* = 0.001 *	r = 0.182, *p* = 0.12
OGTT-2’	r = 0.19, *p* = 0.105	r = 0.168, *p* = 0.152
Delivery BMI (kg/m^2^)	r = 0.418, *p* < 0.001 *	r = −0.06, *p* = 0.61
BMI—2 day (kg/m^2^)	r = 0.373, *p* = 0.001 *	r = −0.03, *p* = 0.798
Weight—2 day (kg)	r = 0.456, *p* < 0.001 *	r = −0.08, *p* = 0.498
LTI (kg/m^2^)	r = −0.022, *p* = 0.853	r = −0.193, *p* = 0.1
FTI (kg/m^2^)	r = 0.353, *p* = 0.002 *	r = 0.021, *p* = 0.862
TBW (%)	r = 0.424, *p* < 0.001 *	r = −0.132, *p* = 0.263
E/I	r = 0.446, *p* < 0.001 *	r = 0.01, *p* = 0.931
BCM (kg)	r = 0.078, *p* = 0.506	r = −0.133, *p* = 0.258

*p*—Qualitative variables: r—Spearman’s correlation coefficient; *—difference statistically significant (*p* < 0.05). HDL—high-density lipoprotein cholesterol; LDL—low-density lipoprotein cholesterol; HbA1c—hemoglobin A1c; MCV—mean corpuscular volume; OGTT—oral glucose tolerance test; BMI—body mass index; LTI—lean tissue index; FTI—fat tissue index; TBW—total body water; E/I—extracellular water to intracellular water index; BCM—body cell mass.

## Data Availability

The data presented in this study are available on request from the corresponding author.
